# Safety analysis of brachial artery sheath removal after heparin reversal with a half dose of protamine after percutaneous coronary intervention: a single-center experience

**DOI:** 10.3389/fcvm.2024.1479506

**Published:** 2025-01-06

**Authors:** Huanhuan Wang, Cheng Cui, Dan Liu, Hongmei Liu, Tao Tian, Minghao Liu, Bo Zhang, Tongqiang Zou, Zhan Gao, Lijian Gao, Haibo Liu

**Affiliations:** ^1^ Department of Cardiology, National Center for Cardiovascular Diseases, Fuwai Hospital, Chinese Academy of Medical Sciences, Beijing, China; ^2^ Department of Cardiology, Shihezi People’s Hospital, The Third Affiliated Hospital of Shihezi University School of Medicine, Xinjiang, China; ^3^ Department of Cardiology, Yunnan Fuwai Cardiovascular Hospital, Kunming, Yunnan, China

**Keywords:** brachial artery, heparin, protamine, percutaneous coronary intervention, safety

## Abstract

**Aim:**

To evaluate the safety of brachial artery (BA) sheath removal after heparin neutralization with a half dose of protamine immediately after percutaneous coronary intervention (PCI).

**Methods:**

The clinical data of 209 consecutive patients who underwent PCI through the BA at Fu Wai Hospital between September 2019 and June 2024 were retrospectively collected. In group I, the brachial sheath was removed 4 h after the PCI procedure. In group II, circulating heparin was neutralized with a half dose of protamine sulfate, and the brachial sheath was removed immediately after the procedure.

**Results:**

There were no cases of acute stent thrombosis, nonfatal myocardial infarction or in-hospital mortality in either group. In group II, there were two cases of pseudoaneurysm, one of which was transfer to surgery and the other was manually compressed. No severe puncture site-related bleeding occurred. The levels of hemoglobin were similar between the two groups before and after the PCI procedure (*p* > 0.05).

**Conclusions:**

The BA sheath can be safely removed immediately after PCI by neutralizing heparin with a half dose of protamine. But we still need to be vigilant about the occurrence of pseudoaneurysms.

## Introduction

Coronary artery disease (CAD) is the leading cause of morbidity and mortality worldwide (1). Patients who undergo coronary catheterization (CC) via transradial access (TRA) are less likely to experience complications related to the access site and discomfort while walking in the early post-procedure period (2). According to the latest ESC guidelines, transradial access is recommended as the standard method for CC (3). However, TRA is also associated with several complications, such as hematoma, arteriovenous fistula, pseudoaneurysm, osteofascial compartment syndrome, and radial artery occlusion (RAO) (4). For patients with contraindications to puncture of the radial artery, routine puncture of the femoral artery (FA) is recommended. However, FA access may have more serious complications, such as retroperitoneal hematoma and pseudoaneurysm, as well as the need for bed rest after the procedure. FA puncture increases hospitalization time and is also uncomfortable for patients.

On occasion, neither the RA nor the FA can be safely accessed, such as in patients with severe peripheral vascular disease, an impalpable RA or unsuitable FA. Thus, a percutaneous brachial approach is often used in these patients.

In clinical practice, puncture of the BA often delays extubation to allow an activated clotting time (ACT) within 4 h after the percutanous coronary interventin (PCI) procedure. According to the literature, patients can be immediately and safely extubated after the administration of protamine (5). Therefore, the purpose of this study was to retrospectively analyze the safety of immediate BA sheath removal after heparin reversal with a half dose of protamine after PCI.

## Materials and methods

### Study population

A retrospective observational study of 215 continuously enrolled patients who underwent PCI through the BA at Fu Wai Hospital between September 2019 and June 2024 was performed. Six patients who received bivalirudin for anticoagulation therapy during the procedure were excluded. The remaining patients were divided into two groups: group I, which underwent brachial sheath removal 4 h after the PCI procedure without ACT, and group II, which underwent brachial sheath removal immediately after circulating heparin was neutralized with a half dose of protamine sulfate.

### Procedural details

**Figure 1:**
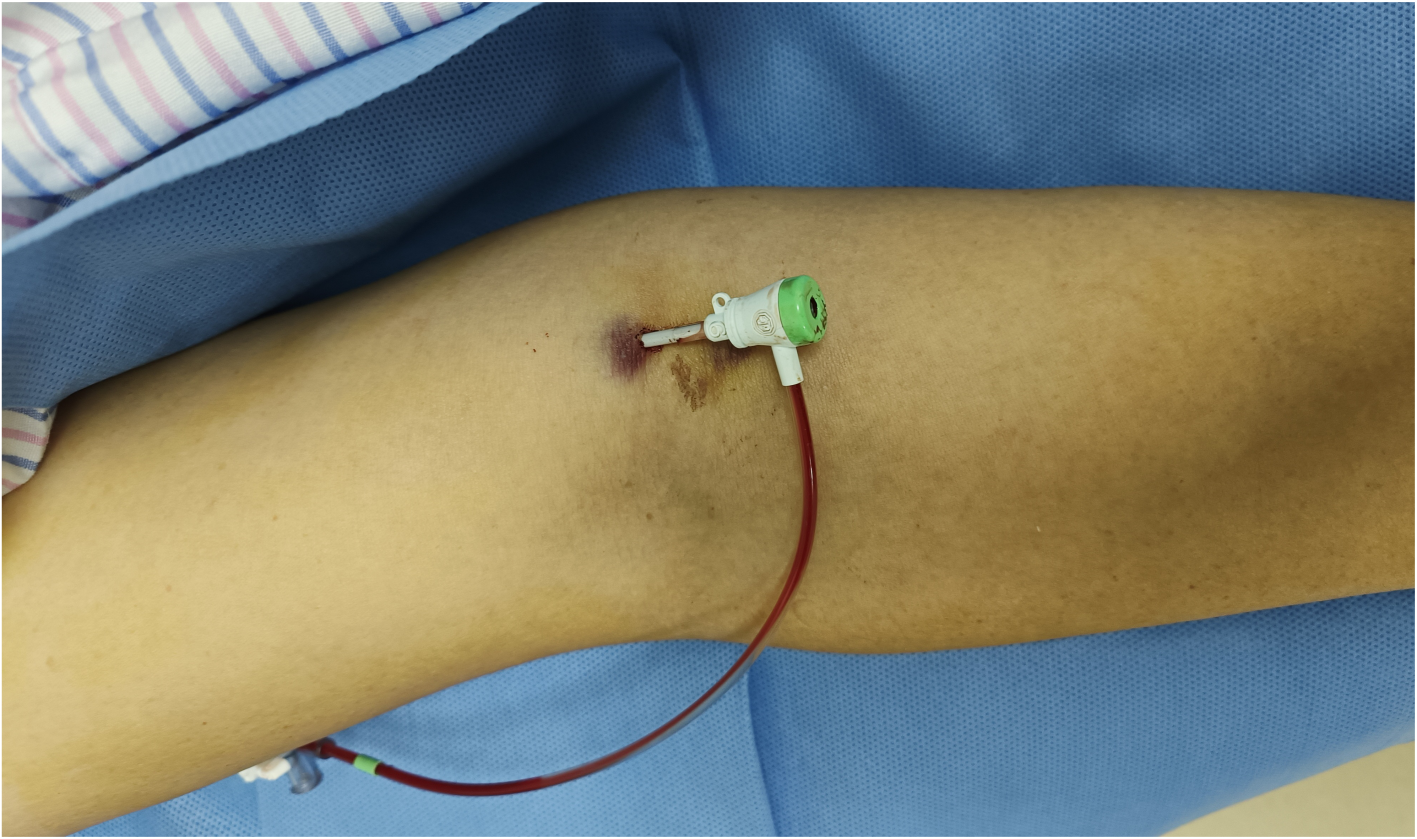
Implantation of sheath after brachial artery puncture.

**Figure 2:**
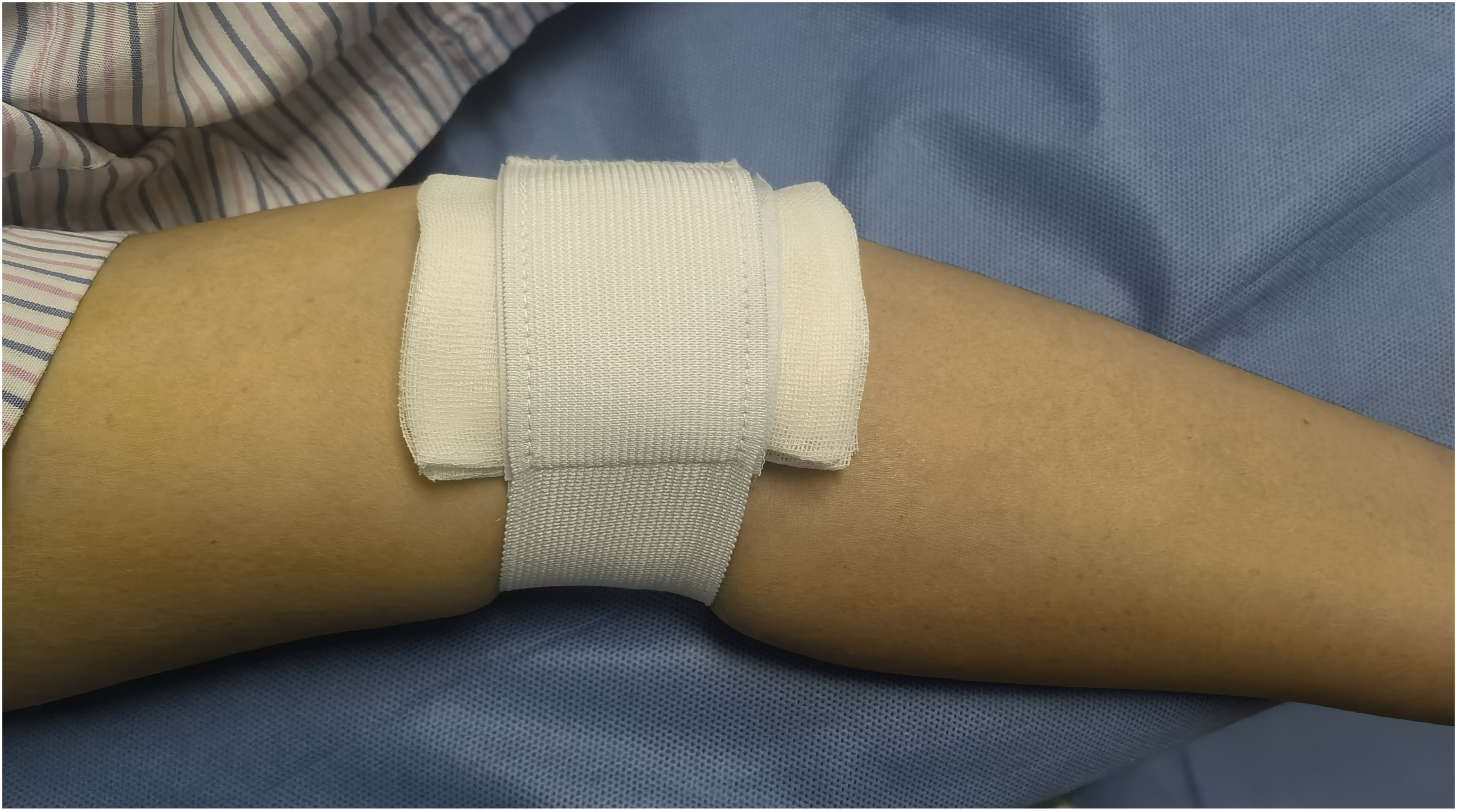
Wrap with elastic bandage at the puncture site of the brachial artery to stop bleeding.

Before the procedure, all patients received sufficient oral doses of dual antiplatelet drugs (aspirin + clopidogrel or aspirin + ticagrelor). The patient was positioned flat on the bed with their palms facing up, and the puncture point was located at the strongest pulsation point on the inner lower one-third of the upper arm, 2 cm above the skin folds on the elbow. Local anesthesia with 2% lidocaine was applied to the puncture site, and the modified Seldinger method was used for nontransmural vascular puncture and extubation (Figure 1). During the procedure, unfractionated heparin (100 U/kg) was administered to all patients, and the use of glycoprotein IIb/IIIa inhibitors was based on the operator's judgment. The PCI strategy and stent type were selected by the treating physician. In group I, the BA sheath was removed in the ward by the cardiology resident four hours after the PCI procedure without assessing the ACT. In group II, according to the dosage of heparin, a half dose of protamine sulfate was administered immediately after the procedure. The sheath was subsequently removed by the operator who performed the PCI. After 15 min of local compression with elastic bandages (Figure 2), the patient could ambulate immediately.

### Endpoints and definitions

The primary endpoints were in-hospital death, acute stent thrombosis (ST) and major bleeding complications. Myocardial infarction (MI) was defined according to the third universal definition of MI (6). ST was defined on the basis of the Academic Research Consortium definitions, and the level of certainty was regarded as definite or probable (7). Major bleeding was defined in accordance with the Bleeding Academic Research Consortium definitions and categorized into grades 3–5 (8). All endpoints were adjudicated centrally by two independent cardiologists, and any disagreements were resolved by consensus.

### Statistical analysis

Statistical analysis was performed using SPSS 23.0 statistical software. The measurement data are presented as means ± standard deviations (*x* ± s), and the Categorical variables are expressed as a percentages (%). A *P*-value < .05 was considered statistically significant.

## Results

### Baseline patient characteristics

The baseline characteristics of the study population are shown in Table 1. A total of 209 patients were included, and 104 patients were included in group I and 105 patients in group II. In Group II, there is a higher proportion of female patients(26% vs. 41%, *p* = 0.022). There was no significant difference in age, coexisting conditions, clinical presentation, or concomitant medication use between the two groups of patients.

**Table 1:** Clinical characteristics.

	Group I	Group II	*P*-value
	(*n* = 104)	(*n* = 105)	
Demographic characteristics			
Age; years	63 ± 6	60 ± 6	0.598
Female gender (%)	27 (26.0)	43 (41.0)	0.022
Co-existing conditions (%)			
Hypertension	57 (54.8)	69 (65.7)	0.107
T2DM	34 (32.7)	36 (34.3)	0.807
Dyslipidemia	64 (61.5)	69 (65.7)	0.530
Family history	5 (4.8)	9 (8.6)	0.276
Previous MI	29 (27.9)	21 (20.0)	0.182
Previous PCI	47 (45.2)	42 (40.0)	0.448
Previous CABG	7 (6.7)	6 (5.7)	0.761
CVD	11 (10.6)	10 (9.5)	0.800
PAD	6 (5.8)	4 (3.8)	0.507
Clinical presentation (%)			
ACS	62 (59.6)	61 (58.1)	0.823
Stable angina	34 (32.7)	35 (33.3)	0.922
Silent ischemia	6 (5.8)	7 (6.7)	0.788
Medication at discharge (%)			
Aspirin	102 (98.1)	102 (97.1)	0.659
Clopidogrel	80 (76.9)	88 (83.8)	0.210
Ticagrelor	23 (22.1)	12 (11.4)	0.039
Beta blocker	88 (84.6)	87 (82.9)	0.731
ACEI/ARB	59 (56.7)	59 (56.2)	0.937
Statin	104 (100.0)	102 (97.1)	0.083
Ezetimibe	60 (57.7)	65 (61.9)	0.535
PPI	59(56.7)	61(58.1)	0.842

T2DM, type 2diabetes mellitus; MI, myocardial infarction; PCI, percutaneous coronary intervention; CABG, coronary artery bypass grafting; CVD, cerebral vascular disease; PAD, peripheral vascular disease; ACS, acute coronary syndrome; ACEI, angiotensin-converting enzyme inhibitor; ARB, angiotensin receptor blockade; PPI, proton pump inhibitor.

Data are expressed as mean ± standard deviation; or counts (percentage).

### Procedural characteristics

There was no significant difference in the type of intervention treatment between the two groups (emergency or elective PCI). There was no significant difference between the two groups of patients in terms of lesion type, target vessel intervention, number of drug boluses used, number of stents used, or proportion of f patients who underwent intravascular ultrasound (*p* > 0.05) (Table 2).

**Table 2:** Procedural characteristics and in-hospital complications.

	Group I	Group II	*P*-value
	(*n* = 104)	(*n* = 105)	
Procedural characteristics			
Emergency PCI (%)	4 (3.8)	3 (2.9)	0.691
Elective PCI (%)	100 (96.2)	102 (97.1)	0.691
Target vessel (%)			
LM	10 (9.6)	5 (4.8)	0.174
LAD	49 (47.1)	44 (41.9)	0.449
LCX	36 (34.6)	27 (25.7)	0.161
RCA	30 (28.8)	32 (30.5)	0.796
SVG	2 (1.9)	2 (1.9)	0.992
Type B2/C lesion (%)			
A	9 (8.7)	11 (10.5)	0.654
B1	13 (12.5)	13 (12.4)	0.979
B2	31 (29.8)	26 (24.8)	0.413
C	57 (55.9)	56 (53.8)	0.769
CTO (%)	15 (14.4)	17 (16.2)	0.723
IVUS application (%)	13 (12.5)	9 (8.6)	0.355
DCB (%)	41 (39.4)	42 (40.0)	0.932
Number of stents	1.2 ± 1.0	0.8 ± 1.0	0.862
Average diameter (mm)	2.27 ± 0.39	2.66 ± 0.38	0.135
Average length (mm)	21.8 ± 7.4	22.1 ± 8.4	0.139
Duration of PCI procedure (min)	44.0 ± 23.0	38.9 ± 20.3	0.553
Dosage of contrast (ml)	175.2 ± 32.6	177.0 ± 46.2	0.177
Heparin and protamine dosage			
Average weight (kg)	73.0 ± 13.0	71.0 ± 12.7	0.846
Heparin dosage (mg)	71.7 ± 13.0	70.8 ± 12.7	0.901
Protamine dosage (mg)	–	34.9 ± 8.8	–
In-hospital complications			
Puncture site hematoma (%)	0 (0)	1 (1.0)	0.318
Severe bleeding (%)
HB before PCI (g/L)	144.3 ± 17.1	142.9 ± 15.7	0.422
Hematocrit before PCI (%)	43.0 ± 5.0	43.2 ± 5.6	0.547
HB after PCI (g/L)	131.2 ± 15.5	129.2 ± 16.0	0.751
Hematocrit after PCI (%)	39.2 ± 4.6	38.9 ± 6.0	0.483
Pseudoaneurysm (%)	0 (0)	2 (1.9)	0.157
		1 transfer to surgery 1 manual compression	
Acute stent thrombosis (%)	0	0	–
In-hospital mortality (%)	0	0	–

PCI, percutaneous coronary intervetion; LM, left main; LAD, left anterior descending artery; IVUS, intravascular ultrasound; LCX, left circumflex artery; RCA, right coronary artery; CTO, chronic total occlusion; DCB, drug coated balloon.

Patients in Group I received an average of 71.7 ± 13.0 mg of heparin. Patients in Group II received an average of 70.8 ± 12.7 mg of heparin and an average of 34.9 ± 8.8 mg of protamine for heparin neutralization immediately after the PCI procedure.

Only one patients had hematoma in group II, neither group of patients experienced severe bleeding, and there was no significant difference in hemoglobin or hematocrit between the two groups before and after the procedure. Two patients had pseudoaneurysm in group II, one patinet transfer to surgery and the other one had manual compression. Neither group of patients experienced acute stent thrombosis nor in-hospital mortality (Table 2).

## Discussion

This paper presents the results of heparin neutralization with a half dose of protamine after PCI and immediate removal of the BA sheath in a large cohort of consecutive, nonselected CAD patients. We found that this strategy was indeed safe and was associated with a very low risk of complications. But we still need to be vigilant about the occurrence of pseudoaneurysms.

Although the radial and femoral arteries are conventional accesses for intervention in most cases, they are also not suitable access sites in for some patients. Owing to extensive intervention through the RA, the incidence of RAO is reportedly between 1% and 10% (9). In addition, the rates of second puncture and intubation failure using the same RA were 3.5% (male) and 7.9% (female), respectively (10). FA puncture is associated with serious complications, such as retroperitoneal hematoma and pseudoaneurysm, and requires bed rest after the procedure, which increases the hospitalization time and increases patient discomfort. Therefore, for patients with contraindications to puncture of the radial and femoral arteries, the BA may be a good choice.

In the early stages of coronary angiography in the 1970s, PCI was performed through the BA. However, owing to the puncture strategies and operating instruments used at that time, once bleeding occurred, the patient would likely develop compartment syndrome of the bone fascia and experience compression of the median nerve, which often led to ischemia in the entire upper limb and subsequent hand disability (11). Earlier studies have shown that the incidence of complications, mainly bleeding complications and pseudoaneurysms, at the BA puncture site ranges from 7%–11% (12), whereas others have shown that the incidence of complications can reach as high as 36% (13). Therefore, its clinical application is greatly limited.

Concerns about using the BA are limited to compression hemostasis. A meta-analysis including fifteen articles published after 2008 revealed the rates of complications rates associated with percutaneous BA interventions. Seventy-five of 1,424 (5.27%) patients experienced major complications at the access site. Thirteen of 309 (4.21%) patients who underwent hemostasis with a vascular closure device (VCD) experienced major complications, and 65 of 1,122 (5.79%) patients who underwent hemostasis with manual compression (MC) experienced major complications. The major access site complication rate associated with TBA was 5.27% (14). In recent years, owing to the extensive use of the BA in peripheral vascular intervention, the BA has once again become a focus of attention for interventional cardiologists. Owing to the development of technologically advanced surgical instruments, the incidence of complications associated with the BA has significantly decreased compared with that reported previously. Recent studies have shown that the BA can be a safe and effective alternative to the FA for access, with complication rates reportedly ranging between 1.3% and 3.4% (15, 16).

Unlike the radial route, there are no VCDs suitable for external arterial compression in China. Some studies have indicated that the BA sheath should not be removed until normal coagulability has been restored (ACT < 180 s) (17), so the BA sheath is usually removed 4 h after the procedure to allow heparin to metabolize, reducing the risk of bleeding, and then MC is used for hemostasis. This method is not very convenient for interventional cardiologists and also increases the workload of ward nurses. Therefore, we aimed to explore the safety of neutralizing heparin with protamine for immediate extubation.

Protamine was used to neutralize circulating heparin was in earlier studies. In 1997, Pan et al. randomly divided 228 consecutive patients whose stent implantation was successful into 2 groups, one of which received 2 mg/kg of protamine and underwent in-laboratory sheath removal and reported that heparin could be safely reversed with protamine immediately after stent implantation (18). Lin et al. consecutively enrolled 105 femoral PCI patients; 78 underwent stent implantation (1.3 ± 1.1 stents), and heparin was reversed with 0.5 mg/100 U of protamine. The ACT was checked before and after protamine administration, with the aim of removing the sheath when the ACT was less than 170 s. The average heparin dose was 5,076 ± 1,746 units, the peak ACT was 269 ± 68 s, and the postprotamine ACT was 165 ± 31 s. The average protamine dose administered was 24 ± 6 mg. No significant adverse events occurred except for a single hematoma that did not require surgical intervention (19). Ducas John et al. consecutively enrolled 429 eligible patients who underwent PCI. After the procedure, if the ACT was at or above 160 s, intravenous protamine was administered for 5 min according to the ACT. If the ACT was between 160 and 200, 15 mg of protamine was administered. If the ACT was between 200 and 250, 20 mg of protamine was administered. If the ACT was between 250 and 300 mg, 25 mg of protamine was administered. Repeated doses of protamine were administered if necessary. If the ACT was less than 160 s, the sheath was removed immediately in the catheterization laboratory, and hemostasis was achieved by manual compression or clamp compression. Minor groin bleeding occurred in six patients. One patient required femoral pseudoaneurysm repair. There were no deaths during the 30-day follow-up period. The results showed that immediate reversal of anticoagulation therapy is safe and feasible for immediate sheath removal after PCI (20). The above studies show that administering different doses of protamine to neutralize heparin after PCI is safe and effective.

This was a retrospective study, and all patients included had contraindications to radial and femoral artery puncture or were unwilling to undergo FA puncture. In this study, the BA sheath was quickly removed after the administration of protamine to neutralize heparin without assessing the ACT; owing to heparin metabolism, a half dose of protamine was given on the basis of experience and previous studies on the FA approach.

In the group II, 2 patients developed pseudoaneurysms, with a probability of 1.9%, which is lower than the reported probability in other literature. Reversal with protamine for early sheath removal in this single-center study appears to be efficacious and safe. The BA is located superficially, easily palpable, and has a thicker diameter, providing a thicker sheath. It is a simple and effective alternative to femoral closure devices and the RA approach to early ambulation after PCI. But we still need to be vigilant about the occurrence of pseudoaneurysms.

To our knowledge, this study is the first to explore the safety and effectiveness of immediate sheath removal after neutralization with protamine after BA puncture. This may be another good strategy for patients with contraindications to puncture of the radial and femoral arteries in clinical practice.

## Limitations

This study was limited by its single-center retrospective nature, small sample size, lack of ultrasound examination, and lack of information regarding whether there were local vascular complications. Patient comfort, length of hospital stay, radiation exposure, and fluoroscopy use were not assessed. The procedures were performed by four cardiologists with varying levels of experience, and there was significant operator variability in both the selection criteria and experience in establishing BA access. A larger multicenter study investigating the safety and efficacy of this strategy is suggested, with the goal of reducing costs and expediting care.

## Conclusion

A half-dose of protamine to reverse heparin for early BA sheath removal in this single-center study appears to be efficacious and safe, with no early adverse cardiac events. But we still need to be vigilant about the occurrence of pseudoaneurysms.

## Data Availability

The datasets presented in this article are not readily available due to privacy and ethical restrictions. Requests to access the datasets should be directed to Lijian Gao, gljxra0104@126.com.
